# Management options and long-term follow-ups for permanent double incisors: six to eleven-year case reports

**DOI:** 10.1186/s12903-024-04000-7

**Published:** 2024-02-13

**Authors:** Betül Şen Yavuz, Batın Ilgıt Sezgin, Barhan Pekel, Bora Korkut, Ali Menteş

**Affiliations:** 1 Department of Pediatric Dentistry, Faculty of Dentistry, Marmara University, Istanbul, Türkiye; 2 Private Practice, Istanbul, Türkiye; 3 Department of Restorative Dentistry, Faculty of Dentistry, Marmara University, Istanbul, Türkiye; 4 Istanbul Galata University, Istanbul, Türkiye

**Keywords:** Concrescence, Double teeth, Fusion, Hemisection, Intentional replantation

## Abstract

**Background:**

Double teeth are dental anomalies that can lead to aesthetic and orthodontic problems.

**Case presentation:**

This report discusses two cases involving the multidisciplinary management of permanent maxillary left lateral incisors fused with a supernumerary tooth in two girls aged 9 and 10. Following intraoral and radiographic examinations, one was diagnosed with fusion, and the other was diagnosed with concrescence. The crown of the fused incisor was separated using a burs and extracted intraorally. The concrescent incisor was separated along its length using a laser and intentionally replanted extraorally. After a 6-year follow-up, no pathological signs were observed in the fused incisor. However, after an 11-year follow-up, external resorption was observed in the concrescent incisor.

**Conclusions:**

Both incisors remained asymptomatic throughout the observation period. This case report highlights two different and effective methods employed to preserve the natural function, form, and aesthetics of double incisors.

**Supplementary Information:**

The online version contains supplementary material available at 10.1186/s12903-024-04000-7.

## Background

Double teeth are rare dental anomalies, often observed as gemination, fusion, and concrescence. They have been reported to occur with a prevalence ranging from 0.1% in permanent dentition to 0.2–1.2% in primary dentition in Caucasians [1]. Gemination refers to an incomplete division of a tooth bud, while fusion involves the union of two separate tooth buds before crown mineralization occurs [2]. In gemination, the double teeth may have separate pulp chambers and root canals [3]. Gemination is commonly associated with hyperdontia in the dental arch, whereas fusion is associated with hypodontia. However, the fusion of a supernumerary tooth with a normal tooth can lead to different results [4, 5]. Concrescence, on the other hand, refers to the union of roots from two different teeth [1].

The etiology of double teeth is not fully understood [1, 5]. Concrescence can occur during tooth development or in cases where there is no functional activity in the periodontal tissues of one or two teeth due to crowding, chronic pulp inflammation, or excessive orthodontic force [1]. The etiology of fusion or gemination may involve autosomal inheritance as well as environmental factors such as hypervitaminosis or fetal alcohol exposure that can cause local trauma or pressure during tooth development [5].

Fusion and gemination are often observed in the maxillary anterior region, while concrescence is more commonly found in the maxillary posterior region [6]. These anomalies can lead to problems of eruption, caries, periodontal, and aesthetic in both primary and permanent dentitions, as well as orthodontic issues such as diastema, malocclusion, and protrusion [3, 5, 7]. When considering treatment options, the patient’s functional, orthodontic, aesthetic, and periodontal needs should be taken into account. Several alternative treatment approaches have been proposed in the literature, ranging from no treatment to extraction of the tooth with anomalies followed by orthodontic closure of the space or bridge [5]. The treatment of these anomalies requires a multidisciplinary approach due to differences in crown and root structures, endodontic challenges, and aesthetic considerations [8].

This report presents the clinical management of two cases involving a fused and a concrescent incisor.

## Case presentations

These case reports were prepared according to the CARE 2013 Guideline.

### Case 1

In 2012, a 10-year-old girl was diagnosed with a supernumerary tooth located adjacent to the maxillary left lateral incisor. The patient had an Angle Class I molar relationship bilaterally and was in the late mixed dentition. The patient’s clinical examination and medical history did not reveal any systemic disease/syndrome or family history. Extraction of the supernumerary tooth had been attempted at another dental clinic but was referred to the Pediatric Dentistry Clinic of Marmara University due to the complete movement of both teeth. Periapical and panoramic radiography confirmed the diagnosis of a fused concrescent left lateral incisor in the maxilla, which had two fused roots with two independent root canals and two pulp chambers. The axial and cross-sectional images of cone-beam computerized tomography (CBCT) revealed the junction between two teeth by cementum and no connection by enamel. The intraoral examination of the 10-year-old male brother of the patient also revealed the presence of a supernumerary right lateral incisor.

**Fig. 1:**
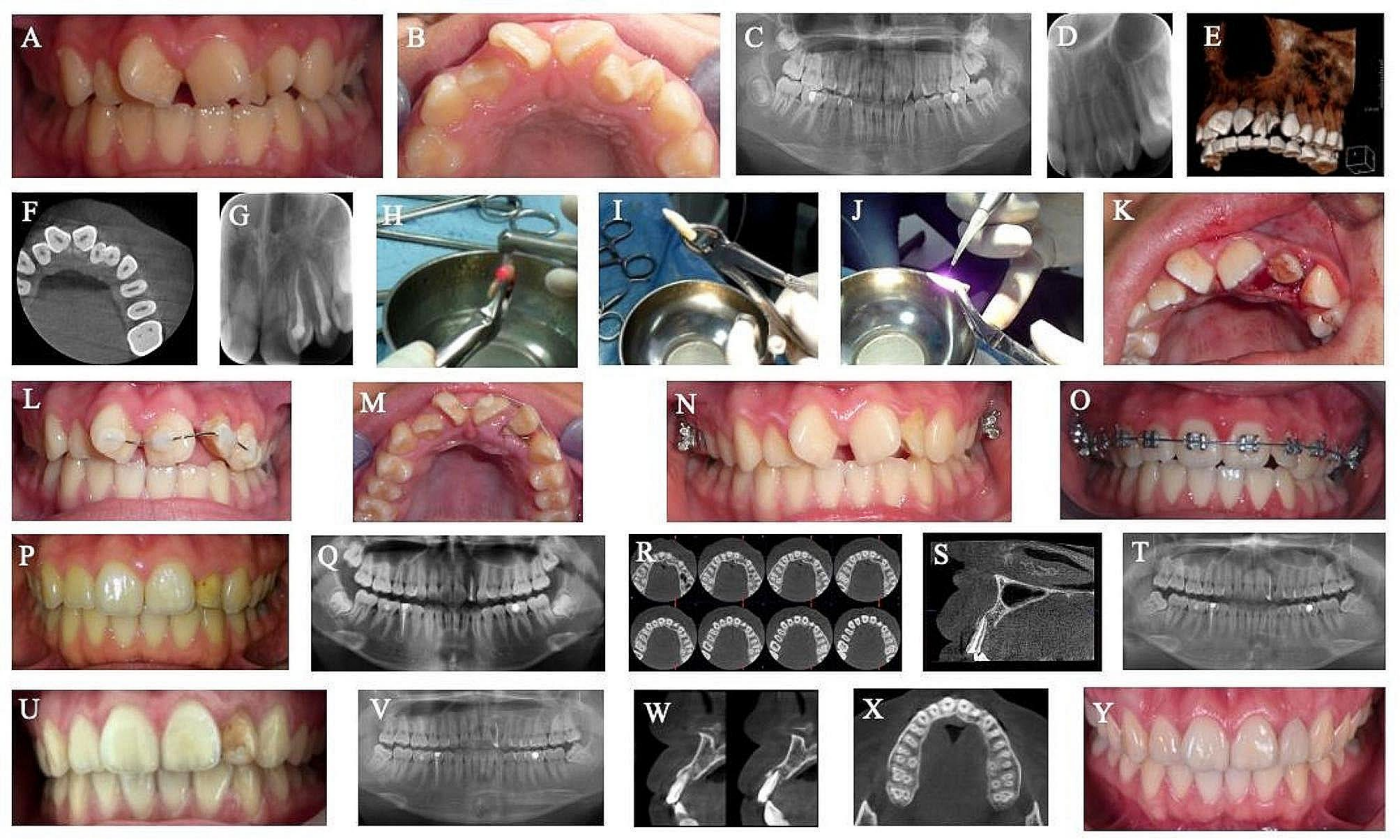
Treatment progress of the 10-year-old girl with a fused left lateral incisor. (**A–D**) pretreatment intra-oral photograph and panoramic and periapical radiographs, (**E–F**) 3-dimensional reconstructed images and coronal views of CBCT, (G) the root canal treatment of double incisor, (**H–K**) the hemisection procedure with Er:YAG laser, Nd:YAG laser application to root surface and replantation of the tooth, (**L–M**) the semi-rigid fixation after the replantation, (**N–O**) Nance appliance and orthodontic alignment, (**P–S**) intraoral photograph, panoramic radiograph, coronal and axial views of CBCT 5 years after the replantation, (**T**) digital radiograph 9 years after replantation, (**U–X**) intraoral photograph, panoramic radiograph, axial and coronal views of CBCT 11 years after replantation, (**Y**) Final intraoral photograph after restoration renewal

The treatment protocol (Fig. 1) was explained, informed consent was prepared for the parents, and assent was obtained from both the parents and the child. The decision on which incisor would remain in the oral cavity was planned by evaluating the root morphologies after the incisors were separated. Therefore, root canal treatment was performed in one session for both teeth after local anesthesia. For each of them, canal lengths were measured, and they were shaped according to the step-back technique. After cleansing and irrigation of the canal, root canal sealer (AH Plus, Dentsply, De Trey, Konstanz, Germany) and gutta-percha (Gutta Perca Points, DiaDent Group International, Korea) were used to fill the canal. Composite resin (Filtek Z 250 Universal, 3 M ESPE, Seefeld, Germany) was placed on both teeth. Double teeth were extracted atraumatically. Following surgical extraction, Er:YAG laser (Fotona Medical Lasers, Fidelis Plus 3 Er:YAG and Nd:YAG Dental Laser, Ljubljana, Slovenia) with a wavelength of 2940 nm (power: 6.00–9.00 W, energy: 300 mJ, frequency: 20 Hz, super-short pulse) and water was used immediately to separate teeth longitudinally throughout the root conjunction line. ND:YAG laser with a wavelength of 1064 nm (power: 1.25 W, frequency: 15 Hz, fibre diameter: 320 μm, micro-short pulse) was applied to the root surface of the tooth to be replanted. One of the teeth was intentionally replanted with a slight digital pressure according to its root and crown shapes. Extraoral time was 15 min. Replantation was followed by a semi-rigid fixation for two weeks. A month later, orthodontic alignment was initiated and completed in 8 months. The patient was scheduled for regular check-ups at 1, 2, 3, 6, 12, 18, and 24 months, followed by annual check-ups until the 5th-year. Then the patient was invited for 9th- and 11th-year follow-ups.

**Fig. 2:**
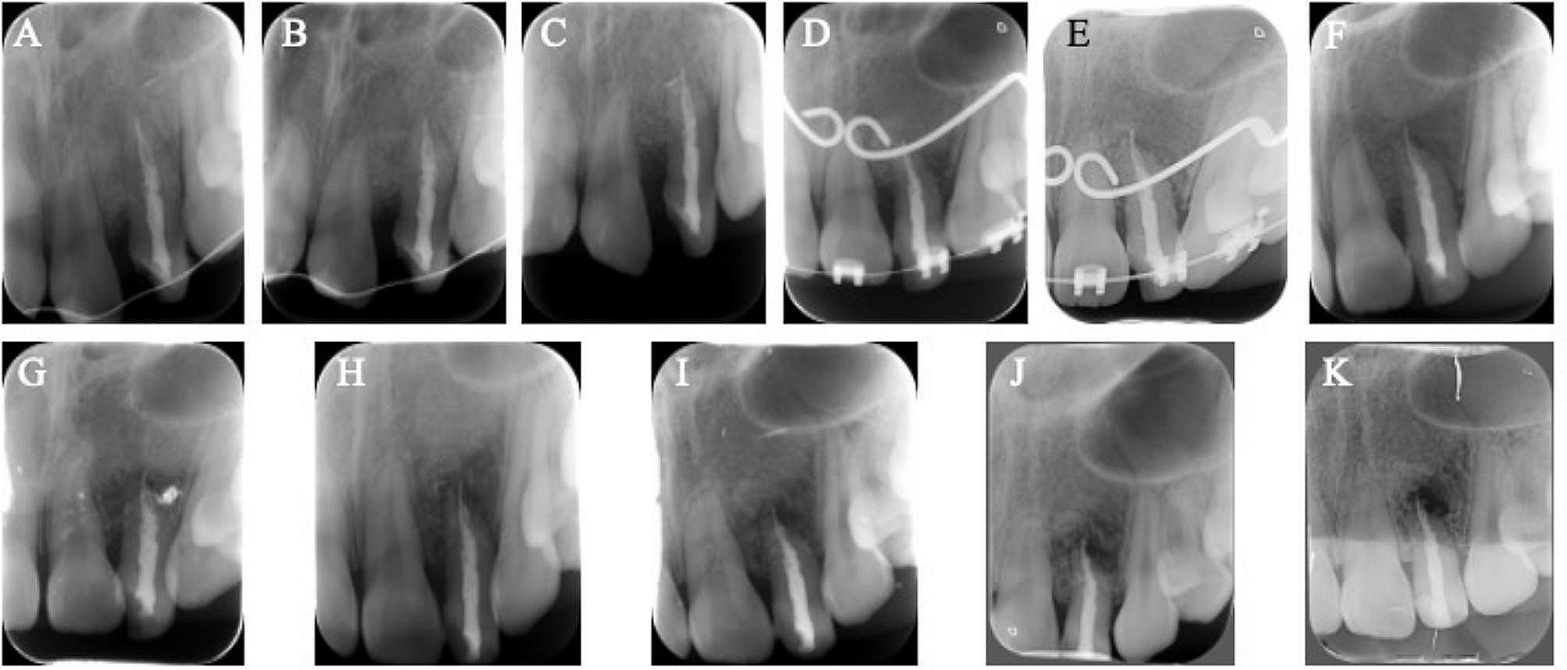
Follow-up periapical radiographs of Case 1 with a fused left lateral incisor. (**A**–**K**) periapical radiographs of 1, 2, 3, 6, 12, 18, 24 months, and 3, 4, 5, and 11 years after replantation, respectively

Follow-up radiographs are shown in Fig. 2. The replanted tooth had no complications after two years of follow-up. In the 3rd-year, external root resorption associated with asymptomatic apical periodontitis was noticed at the apical side of the root. The resorption worsened slightly in subsequent follow-ups. At the 5th-year, CBCT revealed that the root had resorbed still with no obvious clinical symptoms. After eleven years, approximately a quarter of the root was resorbed. The only clinical aspect was discoloration in the restoration. Since external resorption did not progress significantly and showed no clinical symptoms, the decision was made to renew the restoration and continue surveillance for a while longer.

### Case 2

In 2017, a 9-year-old girl was referred due to the unusual appearance of a left lateral incisor and crowding in the premaxilla. The patient had an Angle Class I molar relationship bilaterally and was in the late mixed dentition. The patient was systemically healthy. Clinical and radiological evaluation revealed a T-shaped crown with two separate roots and a fused crown of the lateral incisor with a supplemental tooth. A CBCT was performed to ensure that no additional fusion occurred on the root pulps and root surfaces.

**Fig. 3:**
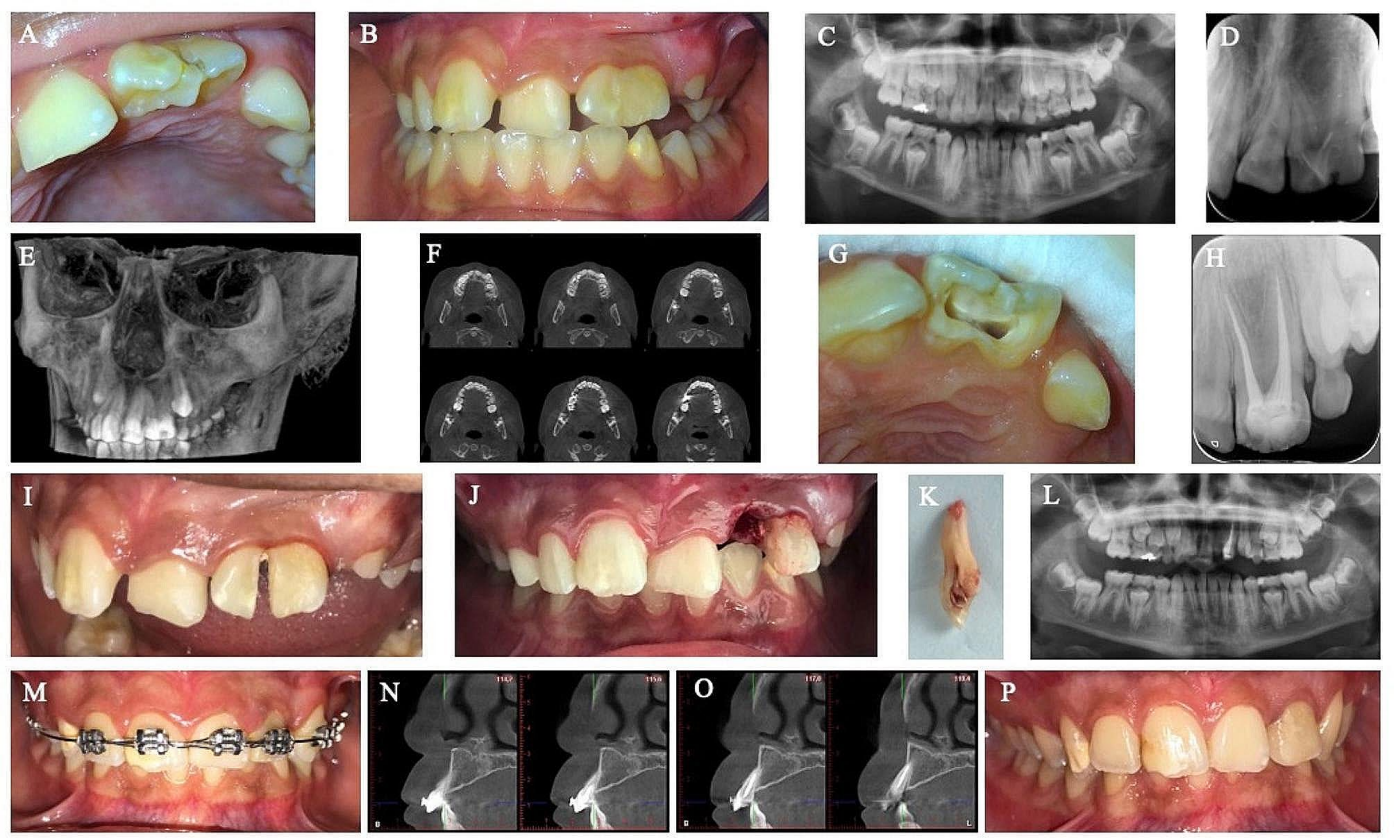
Treatment progress of the 9-year-old girl with a concrescent left lateral incisor. (**A–D**) pretreatment intra-oral photograph and panoramic and periapical radiographs, (**E–F**) 3-dimensional reconstructed images and coronal views of CBCT, (**G–H**) the root canal treatment of double incisor, (**I–K**) the hemisection procedure and extracted tooth, (**L–M**) panoramic radiograph and orthodontic alignment after the hemisection, (**N–O**) the axial CBCT images 18 months after hemisection, (**P**) the intraoral photograph six years after hemisection

**Fig. 4:**
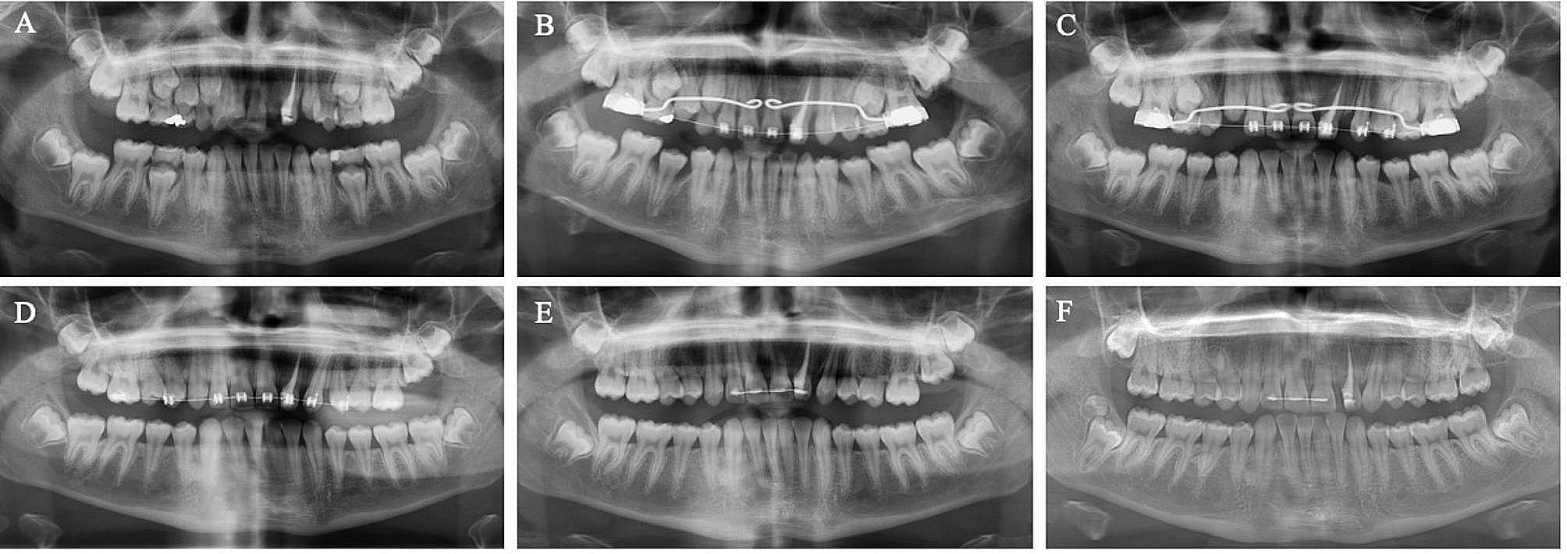
Follow-up panoramic radiographs of Case 2 with a concrescent left lateral incisor. (**A**–**F**) panoramic radiographs of 6, 10, 12, 18, 24 months, and 6 years after hemisection, respectively

The hemisection protocol of the left fused incisors and orthodontic alignment were explained to the patient, and informed consent was obtained. The treatment protocol is presented in Fig. 3. Root canal treatment was initiated, and calcium hydroxide medicament (WP Dental, Hamburg, Germany) was used for dressing. The root canals were obturated with gutta-percha and root canal sealer. Two months later, the crown of the teeth was separated using a fine needle diamond bur, and the mesial part was extracted atraumatically to better preserve the alveolar crest volume and reduce the loss of external contour [9]. The remaining crown was restored with composite resin and the patient was scheduled for three months for recovery. Orthodontic treatment was initiated, and alignment was achieved in 8 months using light forces. The patient was scheduled for regular check-ups at 1, 3, 6, 12, 18, and 24 months. Then, the patient was invited in the 6th-year. Follow-up radiographs are shown in Fig. 4. During orthodontic treatment in the transitional dentition, radiographic evaluations were performed using panoramic radiography at 6-month intervals to evaluate the root angulations of the teeth, dentofacial growth, and tooth/skeletal relationships. In addition to this protocol, an additional panoramic radiograph was obtained in the 10th month to assess the root development of newly erupting permanent teeth at the initiation of orthodontic treatment.

After 1.5 years of treatment, a final CBCT did not show any sign of root resorption. In the 6th-year, there were no clinical or radiographical signs or symptoms of root resorption, pulpal pathology, or ankylosis. However, it was noticed that due to the debonding of the retainer on the left lateral incisor, the inclination of the root was corrected spontaneously, resulting in a diastema mesial to the lateral incisor. The diastema was closed with composite resin. The new restoration was constructed based on the mesiodistal width of the opposing lateral incisor as a reference. In the panoramic radiograph in the 6th-year, new supernumerary teeth between the upper right premolars and distal to the lower right second premolar, and a distomolar tooth in the right mandibular region were observed. A distomolar tooth was suspected in the left maxillary region, and it was decided to reevaluate for diagnosis with further radiographic examinations.

## Discussion and conclusions

Accurate diagnosis and treatment are crucial in managing fusion, gemination, and concrescence. For diagnosis, detecting the presence of hyperdontia or hypodontia in the dental arch is necessary, along with determining the level of fusion [2]. This case report presents two cases with concrescence and fusion merged with supernumerary teeth. There is a hypothesis suggesting that the etiology of these teeth is related to the hyperactivity of the dental lamina [10]. In the second case of this case report, the presence of new supernumerary teeth six years later in the second case supports this hypothesis. However, the presence of a supernumerary tooth in the brother of the first case is also consistent with the finding that etiology is influenced by heredity [5]. The primary reason for concrescence is the absence of space for each tooth to develop individually. If the periodontal ligament activity of an unerupted or infraocclusion tooth is lacking, the interdental bone may atrophy over time and the cementum tissue may approach the root of the other tooth. Initially, the pre-cement tissue of the two teeth, which has not mineralized, combines and mineralizes together. However, if there is dentin tissue in addition to cement tissue at the union site, then it is referred to not as concrescence but as fusion [11]. Although the etiology of the fusion of the double teeth germs is not fully known, autosomal inheritance and physical forces such as trauma were thought to play a role [12]. This local anomaly can also be seen as a secondary symptom of syndromic disorders such as Russel-Silver syndrome, otodental dysplasia, oral-facial-digital syndrome, median cleft facial syndrome, chondroectodermal dysplasia, achondrodysplasia, and focal dermal hypoplasia [12, 13].

These anomalies can lead to orthodontic problems such as tooth crowding, diastema, protrusion, and delayed eruption of adjacent teeth, as well as restorative and aesthetic issues such as dental caries at the fusion line and endodontic treatment due to the progression of caries. After proper diagnosis, optimal treatment requires a multidisciplinary approach and patient cooperation [2]. Various treatments have been reported in the literature for double teeth, such as no treatment, only extraction, orthodontic closure following hemisection or extraction, or post-extraction prosthetic restoration [5, 14–16]. In both current cases, due to severe crowding, one of the double incisors was planned for extraction and orthodontic alignment.

Different options are available for the management of double incisors. In addition to the restoration of dental caries formed in the groove in the fusion line, a prophylactic seal with composite resin without dental caries has also been suggested. The mesiodistal dimension of the crown might be reduced, and if the pulp chamber is bifid, orthodontic alignment is performed after endodontic treatment [4, 17]. Depending on the fusion level, extraoral hemisection and intentional replantation of the tooth could be considered, ensuring sufficient bone preservation for a further implant/prosthesis [2, 7, 17]. Alternatively, extraction of the double teeth, orthodontic closure of the space, or prosthesis/implant could be considered as the last option [17]. Since both double teeth in the current cases had separate pulp chambers, endodontic treatments were performed intraorally. While the fused incisors merged at the coronal level were intraoral hemisected with a burs, the concrescent incisors merged along the cementum surface were extraoral hemisected with a laser. The use of lasers reduces the risk of contamination and does not create debris and smear layers compared to the use of burs. Before replantation, Nd:YAG laser was applied to the root surface to prevent root resorption [18] and increase biocompatibility [19]. However, Friedman et al. [20] stated that the clinical application of surface modification with the Nd:YAG laser is not warranted. Despite the surface modification with Nd:YAG, external resorption was seen in this case, supporting this idea. Moreover, the hemisection procedure was recommended to be performed with multifluted burs, as they create a smoother surface than diamond burs [2]. Steinbock et al. [21] used an osteotome to separate the fused incisors and then applied mineral trioxide aggregate for pulp capping to the remaining tooth. After 10 years, the tooth was still vital, but severe external resorption was observed [21]. In addition, a study also reported that laser application to the socket after extraction not only reduces the bacterial load but also increases epithelialization in the gingival pocket and increases healing [22]. The fact that no laser was applied to the post-extraction socket might be a limitation of the first case.

The intraorally treated fusion showed clinical and radiographic success after 6 years, while intentionally replanted concrescence after extraoral hemisection showed external resorption involving a quarter of the root within 11 years. While the success of intentional replantation varies between 67% and 93%, this rate decreases to 47% in teeth with unsuccessful endodontic treatment [23]. The replantation success is influenced by factors such as endodontic pathology or infection and the extraoral time, which affects the viability of periodontal ligament cells, as well as the type of splint [2]. Despite the formation of resorption in the long-term, replantation preserves the alveolar bone, especially in children and adolescents during the growth and development period, until implant or prosthesis is placed, provides psychological and aesthetic benefits by ensuring the preservation of natural tooth form, and improves the patient’s quality of life [5]. However, a limitation of current cases might be considered that bioactive cements with anti-inflammatory potential, and antimicrobial effect, which provide an appropriate apical seal and promote the proliferation of periodontal ligament cells and alkaline phosphatase activity, were not used as the root canal filling material [2, 24].

The first case in this case report presents an 11-year follow-up of a lateral incisor with concrescence involving a supernumerary tooth. To the best of our knowledge, this is the longest follow-up reported in the literature. In addition, case reports do not have similar evidence levels to randomized clinical trials. However, the fact that the incidence of these anomalies is very low, especially in permanent dentition [1], and that there are many treatment options according to the type of anomaly and level of fusion makes it challenging to conduct single-center randomized studies.

The double incisor, treated intraorally, showed high long-term success. However, in the case of the incisor with fusion at the root level, intentional replantation appeared to be completely successful in the short-term, but external resorption could be expected without any clinical symptoms during long-term follow-up. As a result, it should be noted that the management of double teeth requires a multidisciplinary treatment approach and long-term surveillance.

### Electronic supplementary material

Below is the link to the electronic supplementary material.

Supplementary Material 1

## Data Availability

The datasets used and analyzed during the current study available from the corresponding author on reasonable request.

## Abbreviations

CBCT: Cone-beam computerized tomography
